# EFL learners’ grit, classroom enjoyment and their willingness to communicate: Iranian public school versus private English language institute learners

**DOI:** 10.1186/s40862-022-00150-9

**Published:** 2022-09-15

**Authors:** Faramarz Ebn-Abbasi, Musa Nushi

**Affiliations:** Department of English Language and Literature, Shahid Beheshti University, Tehran, Iran

**Keywords:** Willingness to communicate, Grit, Classroom enjoyment, EFL learners

## Abstract

The pivotal role of communication in second language (L2) learning has triggered plethoric research to identify factors that may influence learners’ willingness to communicate (L2 WTC). However, there is a dearth of comparative research on L2 WTC, especially among EFL learners studying English at different educational institutions. To this end, the present study investigates the role of ‘grit’ and ‘classroom enjoyment’ (CE) in learners’ L2 WTC in two different educational settings of public schools and private language institutes. Grit includes two lower-order constructs, namely perseverance of effort (POE) and consistency of interest (COI), which were examined separately in this study. A total of 269 Iranian students from both public schools and private institutes completed an online survey. What was revealed from the data analysis through the Mann–Whitney u-test, Spearman’s rho, and multiple regression analysis is that private institute learners enjoyed higher levels of WTC compared to public school students. While POE and CE exerted a significant effect on L2 WTC in both educational settings, COI failed to do so. The findings of this study are discussed from a socio-educational perspective with regard to the difference between these two educational contexts.

## Introduction

Success in learning a second language (L2) requires learners’ consistent and meaningful interaction and participation (Gass, 2013). Since the 1980s, encouraging learners’ participation through target language use has been a prominent part of second language acquisition (SLA) pedagogy and research. With the advent of communicative approaches to English language teaching, speaking to learn and learning to speak has gained increasing popularity among L2 scholars (Ducker, 2018). Yet, many researchers have confirmed that learners differ in their level of willingness to communicate in L2 (L2 WTC) (Khajavy et al., 2016). L2 WTC can be defined as readiness to speak or take part in communication when given a chance. In essence, WTC highlights why some individuals seek opportunities to speak while others avoid them and prefer to remain silent (MacIntyre, 2020). L2 WTC can affect the amount of classroom communication and thus plays an instrumental role in L2 learning. Due to the vital role of positive variables in L2 communication (Wang, 2017), the relationship between these variables and L2 WTC has recently turned into a favorite topic of debate among L2 researchers.

With the blooming of positive psychology and the works of educational psychologists such as Schutz et al. (2007), which advocated the importance of both positive and negative emotions in the SLA, more attention has been directed to the role of positive variables in the SLA literature (e.g., Dewaele & Dewaele, 2018; MacIntyre & Mercer, 2014; MacIntyre et al., 2019). Prior to that, negative affective variables such as anxiety mostly overshadowed the literature. As Arnold and Brown (1999) pointed out, “much more attention is given to the question of negative emotions... (one) should not lose sight of the importance of developing the positive” (p. 2).

Grit is a positive internal variable that has not been studied sufficiently in relation to SLA and, of course, L2 WTC, as much as other internal variables such as motivation and language aptitude. Defined as "perseverance and passion for long-term goals" by Duckworth et al., (2007, p. 1087), grit is believed to be a significant predictor of success and performance (Khajavy et al., 2021a, 2021b). Grit can play a crucial role in the SLA since the process requires extended effort (Hakuta et al., 2000), perseverance, and patience (Alamer, 2021).

Lee (2020, p. 2) defines CE as "the extent to which L2 learning classroom is perceived as providing pleasure." Some researchers have suggested that positive emotions in the classroom can increase “the learners’ consciousness of language input and perceptions of linguistic forms, and boost their use of multiple problem-solving strategies” (Jin & Zhang, 2021, p. 949). Recently, many studies have been carried out that focused on the link between CE and L2 WTC in EFL classrooms (e.g., Li et al., 2021; Tahmouresi & Papi, 2021).

Despite the fact that many language studies have investigated the connection between L2 WTC and positive variables (e.g., Dewaele, 2019; Dewaele & Pavelescu, 2021; Khajavy et al., 2021a, 2021b; Lee, 2020; Lee & Lee, 2020), there is still a paucity of comparative research in this area, especially between EFL learners studying English at different educational institutions. This study investigates the relationship between grit and CE, and L2 WTC among EFL learners in private English language institutes and public schools students in Iran from a socio-educational perspective. The educational atmosphere in the mentioned institutes, although within the same geographical context, is profoundly different. While English courses held in Iranian public schools have been criticized for disregarding communication in the target language, EFL private institutes are believed to value communication competence slightly more (Moradkhani & Haghi, 2017). Moreover, unlike learners in many other EFL contexts, Iranian EFL learners find fewer opportunities to communicate with native speakers of English due to socio-political reasons (Rahimi, 2009), a problem that increases the importance of enhancing communication in Iranian foreign classrooms. Given the differences in attitudes toward teaching English in general and communicative competence in particular in these two dominant educational enterprises in Iran, the suggested interaction between context and personality variables in SLA (Sanz, 2005), and the inadequacy of studies in the public schools that examine influential variables with regard to L2 WTC, conducting this study is of critical importance.

Given the above gaps and to deepen our understanding of L2 WTC and provide pedagogical implications, this study examines the following research questions:

1. Is there a significant difference between the level of L2 WTC of Iranian EFL learners in public schools and private institutes?
2. To what extent do L2 girt (POE & COI) and classroom enjoyment correlate with Iranian EFL public school students’ L2 WTC?
3. To what extent do L2 girt (POE & COI) and classroom enjoyment correlate with Iranian EFL private institute learners’ L2 WTC?

## Literature review

### L2 WTC

For a long time, SLA was dominated by structural approaches, focusing mainly on the mastery of vocabulary and grammar. However, with the blooming of communicative approaches, there has been a significant shift in scholars’ perspectives toward SLA. In this regard, WTC, as an influential aspect of communication in L2, has attracted unprecedented attention from researchers. WTC has its roots in the first language studies and was applied to L2 communication by MacIntyre and Charos (1996). In the beginning, WTC was considered a stable personality variable across time and in different situational contexts. More recently, however, L2 WTC has been presented not only as a stable tendency toward communication, but also as a situational trait. Nowadays, researchers believe that both internal variables, such as L2 motivation and attitude toward L2, and external variables, such as inter-group climate and social support, can influence learners’ L2 WTC (Zhang et al., 2020).

**Fig. 1:**
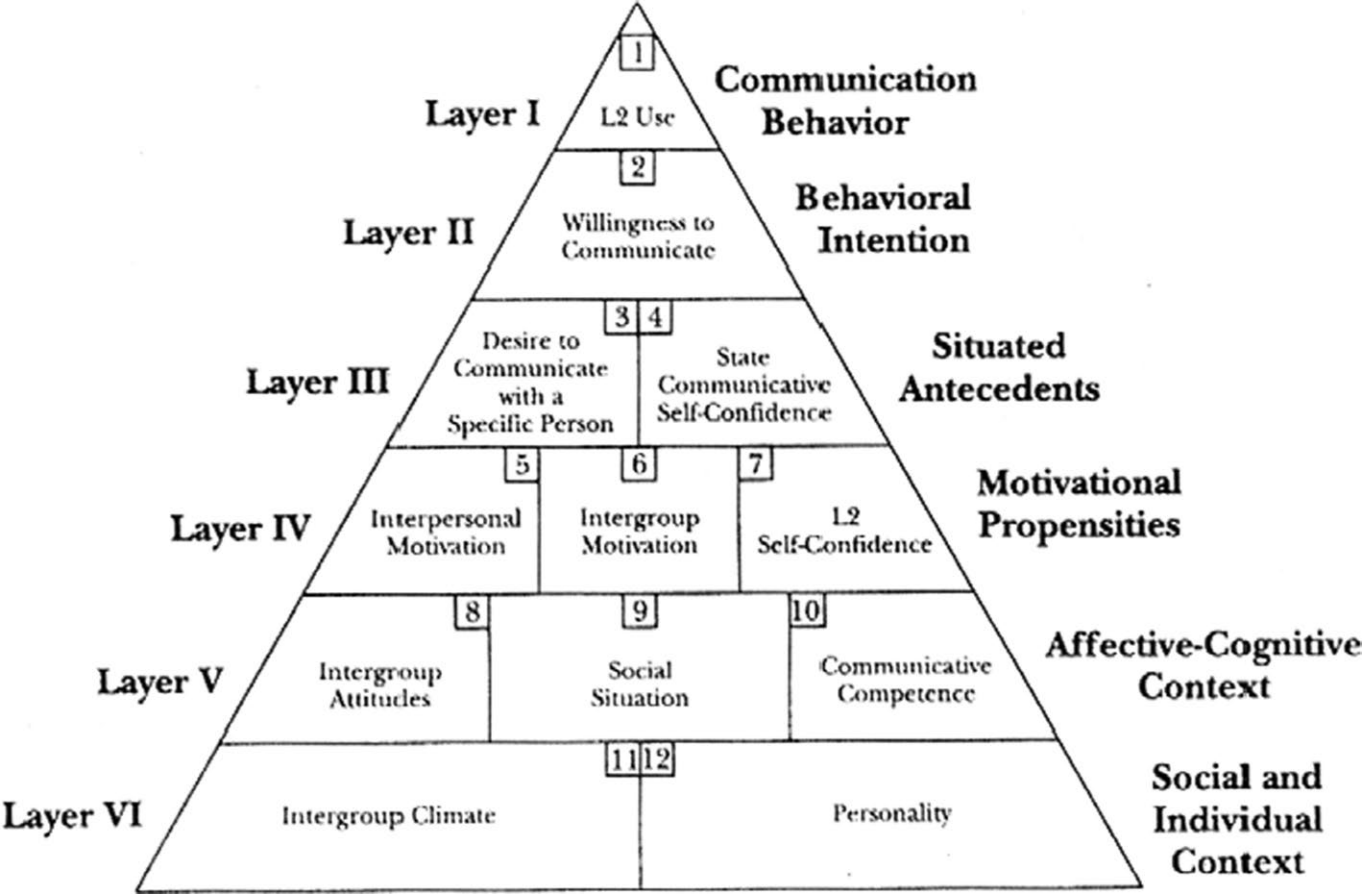
Heuristic model of WTC in L2 by MacIntyre et al., (1998, p. 547)

As the pioneers of L2 WTC, MacIntyre et al. (1998) proposed a groundbreaking model of L2 WTC known as the Heuristic model of WTC in L2 (Fig. 1), which emphasized the complex interactions of varying factors such as context, personality traits, cognition, and emotion. This model can illustrate the multifaceted effects of different variables (individual and situational), including the variables of interest to the present study (i.e., grit and CE), in each layer on the top of the pyramid (i.e., L2 communication). This model consists of six layers which include 12 constructs. In this model, the top layers (I, II, & III) include “more changeable, and context-dependent” (state) variables, and at the bottom layers of the pyramid (IV, V, & VI), more “stable social individual context” (trait) variables are placed (Dewaele & Pavelescu, 2021, p.3). Following this line of research, many researchers have adopted this model to examine the relationship between L2 WTC with a plethora of variables, including motivation (Wu & Lin, 2014), language mindset (Zarrinabadi et al., 2021), anxiety (Zhou et al., 2020), enjoyment (Lee, 2020), and L2 motivational self-system (Lee & Lee, 2020).

### L2 grit

Unlike cognitive skills, non-cognitive skills are relatively new to the SLA field. The scarcity of studies in the literature about non-cognitive skills can be rooted in the intangible nature of these skills that may cause a problematic process of measuring and quantifying (Teimouri et al., 2020). In recent years, however, non-cognitive skills have garnered vast research attention in the SLA domain. Grit as a non-cognitive variable highlights the differences between students’ achievements with the same intellectual talent. Researchers have established that grittier students succeed in achieving higher scores (Khajavy et al., 2021a, 2021b; Strayhorn, 2014), employ persistent effort during learning an L2 (Lake, 2013), and are more likely to experience positive classroom emotions (Wei et al., 2019). Since grit is not a fixed personality trait and can be counted as malleable and teachable, the importance of measuring and estimating grit is gaining unprecedented attention. Duckworth et al. (2007) view grit as a domain-general variable that could be applied to different fields. Teimouri et al. (2020) extended the concept to L2 learning and developed an L2 domain-specific grit notion. They explained that grit as a higher-order factor consisting of two lower-order elements, namely POE and COI, should be examined separately.

### Classroom enjoyment

As the external positive variable, the concept of CE, first developed by Dewaele and MacIntyre (2014), is employed in this study. Boudreau et al. (2018, p. 153) claim that enjoyment is different from routine experiences of pleasure, since “if pleasure can occur simply by performing an activity or completing an action, enjoyment takes on additional dimensions such as an intellectual focus, heightened attention, and optimal challenge.” Unlike pleasure, CE focuses on more permanent feelings of joy in the classroom (e.g., In our EFL classroom, I’m a worthy member of the class). Many researchers have established the CE in relation to L2 WTC as a unique variable different from temporary classroom pleasure (e.g., Lee, 2020).

### Public schools versus private English language institutes in Iran

The present research was conducted in Iran, where Persian is the official language and medium of communication, and English is taught as a foreign language (FL). English finds its way into the country’s school curriculum from the 7th grade as a mandatory subject and is therefore studied by anyone who attends school. Since Iranians hold a positive attitude toward English (Abdolahzadeh & Nia, 2014; Rezaei et al., 2019), it is also taught and learned outside public school contexts (e.g., private English institutes).

The centralized English courses taught in public schools have been criticized wieldy by many language researchers. Some of these criticisms are due to employment of inappropriate methodology, use of low-quality coursebooks, highly crowded classrooms, lack of necessary equipment, inefficient teachers, and failure to achieve the proposed objectives, including helping students to acquire basic communicative proficiency and learn English at a survival level (Farhady & Hedayati, 2009; Moradkhani & Shirazizadeh, 2017; Rahimi, 2009). On the other hand, private English institutes in Iran employ different policies toward language learning. These institutes have been established to promote language use among the learners so that by offering better content and methodology than those in public schools.

FL instruction provided in private centers is fundamentally different from public schools in several ways (Moradkhani & Haghi, 2017). From a curriculum perspective, the ministry of education in Iran has published certain books and assigned the same syllabus for public schools across the country. Khoshsima and Hashemi Toroujeni (2017) revealed that the Iranian public educational system primarily uses Grammar Translation Method (GTM) as its teaching methodology. For many decades, the methodology of such schools consisted of “reading, translation, memorization, and grammar” (Hosseini Goodrich, 2020, p. 9). This approach was replaced with the Communicative Language Teaching (CLT) in 2013; nonetheless, criticism was still directed at the quality and effectiveness of the new English teaching method and its concomitant textbooks (Hosseini Goodrich, 2020). More specifically, the course materials taught at high school are still focused on reading comprehension, grammar, and vocabulary development (Sadeghi & Richards, 2016). However, by employing a decentralized system, private institutes utilize various international coursebooks and different approaches to teaching English. CLT principles are the favorite choice to design private institutes’ curricula, indicating that more attention is given to communication than public schools. These institutes are commonly better equipped and allow fewer students to attend their classrooms compared to public schools. In these institutes, teachers are fortified to encourage communication in the target language. They are required to attend teacher training courses and are mostly assessed via interviews before being given the job (Moradkhani & Haghi, 2017).

### Previous studies

Khajavy et al., (2018) investigated the effects of FL learners’ emotions and positive classroom environment in relation to L2 WTC. They studied WTC with regard to the effects of specific positive and negative emotions, namely enjoyment and anxiety. To this end, they employed doubly latent multilevel analysis as a qualitative methodology. They found that at an individual level both positive (CE) and negative (anxiety) variables influenced L2 WTC, suggesting that L2 learners who experience CE and less anxiety while learning an L2 are more likely to initiate or participate in the act of communication. At a classroom level, Khajavy et al. (2018) observed a relationship between CE and WTC that is equal at both individual and classroom levels. However, this relationship was observed only at the individual level in the anxiety case. It is worth mentioning that the correlation between positive emotions and WTC was stronger and more consistent than that found for negative emotions.

The findings of Khajavy et al.’s (2018) study align well with those of Dewaele et al.’s (2019) study conducted in a different context. They confirmed that FL anxiety and CE are influential negative and positive predictors of L2 WTC, respectively. By employing a multiple regression analysis on data gathered from Spanish students, these researchers suggested frequency of use can also create a sense of WTC among students.

Regarding grit and SLA, Karlen et al. (2019) investigated the role of implicit theories (beliefs regarding whether or not intelligence can be expanded) about self-regulated learning on learners’ (N = 1215) grit as a two-factor model (POE and COI), goal achievement, challenging academic tasks achievement, and learning motivation. They also examined the relationship between POE and COI and learners’ goal achievement, intrinsic and extrinsic motivation, and academic achievement. To this end, these researchers employed a longitudinal self-reports design in the Switzerland. In a nutshell, Karlen et al. (2019) concluded that incremental theory (beliefs that intelligence is a malleable trait that can be improved through hard work) is highly related to grit and adaptive motivational patterns. Interestingly, they observed a weak indirect correlation between POE and academic achievement, while COI showed none. Furthermore, different motivational correlation patterns were obtained between POE and COI to achievement goals and intrinsic motivation in this study.

Wei et al. (2019) observed a direct positive link between grit, foreign language proficiency (FLP), classroom environment, and foreign language enjoyment (FLE). Believing that the level of English among middle school students is still low, while their classroom anxiety is high, the researchers realized that by employing consistent effort into FL learning, students could increase their FL self-efficacy. This will lead to overcoming challenges and setbacks in their path to success. Their study also indicated that grit positively influences FLP through CE and FLE. The researchers explained that one of the reasons beyond this finding can be Asian students' lack of interest in learning another language. Therefore, grit can be created through having a positive environment and enjoyable atmosphere in the classroom to attract students’ interest, which can lead to putting more effort into learning and achieving better FLP. Another finding of the study was the moderating effect of CE between grit and FLE, and also between grit and FLP. Simple slope test results showed that when CE was good, with the increase of grit, the students’ FLE and FLP also significantly increased. However, when CE was poor, the increase of grit did not significantly affect FLE and FLP. This shows that although grit is a positive personality trait, without good CE, its effect on FLE and FLP is minimized. A possible explanation for this could be the effect of environmental factors.

Lee (2020) examined the effects of grit and CE on Korean FL students' WTC. To obtain the data, he utilized Duckworth et al.’s (2007) grit scale, Dewaele and MacIntyre’s (2014) FLE scale, and Lee et al. (2020) five-item WTC scale. Lee realized that POE along with CE correlates positively with L2 WTC among the participants. He explained that students with higher levels of POE who experience a positive environment in the classroom are more likely to show higher levels of WTC. COI, on the other hand, was not observed as a significant predictor of L2 WTC. Another significant finding of this study was the positive relationship between L2 WTC and the length of time devoted to learning English. In other words, students with more experience learning English tend to communicate more compared with others; on the contrary, Lee found out that there is a negative correlation between the age of students and their level of L2 WTC. University students showed lower levels of WTC compared with those students in lower levels of education.

In a recent case study by Dewaele and Pavelescu (2021), the link between FLE, foreign language classroom anxiety (FLCA), and WTC among two high school students of FLE in Romania was examined. The qualitative data collection procedure included observations, a written task, and semi-structured interviews with these students. The analysis of the obtained data revealed FLE and FLCA affect L2 WTC in different ways. An important finding of this study was the direct and indirect influences of previous experiences of using English in/outside the classroom and different personalities of the students on their classroom emotions and WTC in English. Observing a direct link between FLE and FLCA and students' L2 WTC during the interviews and observations, these researchers found out that infrequent participation cannot be taken as a sign of low levels of WTC. It can be a normal response to the context, uninteresting topics, and presumed responses from their classmates.

## Method

### Participants

A total of 269 Iranian EFL learners from both public schools and private institutes were surveyed in this study. The participants were selected based on convenience sampling. Participants from the school were studying in the 11th and 12th grades at the time. As for the institute sample, language learners with elementary (A1) and pre-intermediate (A2) levels of English were selected for the study. These levels were based on the common European framework of reference for languages (CEFR) which describes language ability on a six-point scale, from A1 for beginners, to C2 for advanced students. Gender was not considered a variable in our study. Table 1 represents the demographic characteristics of each group that took part in this study.

**Table 1:** The participants’ demographic information

		Public school (N = 148)	Private institute (N = 121)
Gender	Male	81	59
	Female	67	62
	Total	148	121
Educational stage		11th G* (53%) 12th G (47%)	A1* (42%) A2* (58%)
Age	Mean	17.01	16.39

G = Grade, A1 = Elementary, A2 = Pre-intermediate

### Instruments

The data for each of the variables in the present study were collected using an online survey consisting of three different questionnaires, namely L2 WTC questionnaire (Peng & Woodrow, 2010), grit questionnaire (Teimouri et al., 2020), and CE questionnaire (Dewaele & MacIntyre, 2014). The survey started with a section that elicited the participants’ demographic information. To avoid some extraneous variables, the researchers added two items to the public schools’ questionnaire version. The first item was to detect and remove those public school students who were simultaneously attending private institutes, and the second item was to identify students’ grades. The the questionnaire administered to the learners in the private institutes contained an additional item to detect learners’ current English proficiency level. Moreover, to prevent age differences from affecting our results, the data collected from those institute learners who were older than 18 and younger than 15 were excluded from the analysis. The details of the three questionnaires comprising the survey are detailed below.

#### The L2 WTC questionnaire

A modified version of L2 WTC by Weaver (2005) adopted by Peng and Woodrow (2010) was used in this study (N = 10). The participants answered the questions such as *‘I am willing to ask my group mates in English the meaning of words I do not know’* on a 5-point Likert scale ranging from 1 (definitely not willing) to 5 (definitely willing). The Cronbach’s α for this scale in Peng and Woodrow (2010) was 0.85.

#### The grit questionnaire

Teimouri et al. (2020) developed an L2 domain-specific grit scale that consisted of two sections, namely POE and COI, in the classroom. This questionnaire consisted of 9 items, 5 items were related to POE (e.g., *I am a diligent English language learner*), and 4 items covered COI (e.g., *I think I have lost my interest in learning English*). The participants could select their responses on a 5-point Likert scale ranging from 1 (not like me at all) to 5 (very much like me). The reported Cronbach’s alpha in Teimouri et al.’s (2020) research was 0.80.

#### The classroom enjoyment questionnaire

CE data were collected using a modified version of the original FLE scale developed by Dewaele and MacIntyre (2014). The original questionnaire included 24 items on a 5-item Likert scale (from strongly disagree to strongly agree), from which 16 items (e.g., *I enjoy our EFL classroom*) were chosen that were appropriate for our context. The reported mean of Cronbach’s α for this scale in the Dewaele and MacIntyre’s (2014) study was 0.86.

### Data collection procedure

Google Forms was chosen to host the survey instrument and collect the data. To help the participants comprehend the items better, the questionnaire was translated into Persian by two professional translators. After obtaining the schools (N = 5) and institutes (N = 6) boards’ approval, the link to the survey was sent to the EFL learners.

## Results

### Descriptive statistics

Cronbach’s alpha was used to estimate the reliability of each questionnaire comprising the survey. As seen in Table 2, the questionnaires enjoyed high levels of reliability to elicit WTC, grit, and CE from all the respondents in public schools and private institutes.

**Table 2:** The reliability statistics for the three questionnaires of the survey

Survey sections	Cronbach’s alpha	N
WTC	0.93	10
Grit	0.84	9
POE	0.87	5
COI	0.82	4
CE	0.96	15

For the first research question, the mean and standard deviation for each group were calculated. The results of the descriptive analysis revealed that the mean of L2 WTC in public schools was 2.3, and the standard deviation was 1.16 (Table 3). Table 4 displays the descriptive statistics for L2 grit and CE in public schools. The respondents' mean performance regarding those variables was above 2, which indicates a close to medium performance on L2 grit and CE. The mean of L2 WTC in private institutes was 3.1, and the standard deviation was 1.31, revealing that the participants’ level of L2 WTC was also medium. Table 5 represents the descriptive statistics for POE, COI, and CE among private institutes learners. The mean for POE and COI, which were 2.86 and 3.28, respectively, indicated that the participants' POE and COI were slightly below the medium range. Regarding CE, the learners' responses yielded the highest mean (3.29) in comparison with that obtained for grit (POE & COI) which was also medium (Table 4).

**Table 3:** Means and standard deviations of WTC items

	N	Mean	SE	SD
Public schools	148	2.3	0.13	1.162
Private institutes	121	3.1	0.12	1.31

**Table 4:** Descriptive statistics for L2 grit (POE & COI), and CE in public school students

	N	Mean	SD	Skewness		Kurtosis	
POE	148	2.26	1.26	0.589	0.19	− 0.89	0.39
COI	148	2.44	1.210	0.59	0.19	− 0.69	0.39
CE	148	2.54	1.28	0.43	0.19	− 0.97	0.39

**Table 5:** Descriptive statistics for L2 grit (POE & COI), and CE in private institute learners

	N	Mean	SD	Skewness		Kurtosis	
POE	121	2.86	1.287	0.29	0.22	− 1.04	0.43
COI	121	3.28	1.10	0.45	0.22	− 1.11	0.43
CE	121	3.29	1.22	0.07	0.22	− 1.12	0.43

To investigate how public school students and private institute learners differ in their level of L2 WTC, Kolmogorov–Smirnov and Shapiro–Wilk tests were first conducted to see if the data were normally distributed. The results showed the data did not enjoy normal distribution (Table 6); therefore, following McKnight and Najab’s (2010) suggestion, the Mann–Whitney U test, which assumes no specific distribution, as the nonparametric equivalent of the independent t-test, was employed in this study. As shown in Table 7, the Mann–Whitney U test showed a significant difference between the level of L2 WTC in private and public school learners (U = 7382.500, p = 0.013). The effect size was small (0.151) according to Cohen’s (1992) classification of effect sizes.

**Table 6:** Tests of normality for the variables

	Institutes	Kolmogorov-Smirnov^a^			Shapiro–Wilk		
		Statistic	Df	Sig	Statistic	Df	Sig
WTC total	Private institutes	0.239	121	0.000	0.818	121	0.000
	Public schools	0.293	148	0.000	0.769	148	0.000

^a^Lilliefors significance correction

**Table 7:** Mann–Whitney U test results

Mann–Whitney U	Wilcoxon W	Z	Asymptotic. sig. (2 tailed)
7382.500	18,408.500	− 2.483	0.013

### Correlational analyses

Spearman correlation was used to investigate the relationship between L2 WTC, L2 girt (POE & COI), and CE among the EFL learners in public schools and private institutes (questions two and three). The results of the Spearman correlation revealed that, in public schools, there was a significant positive association between CE and WTC, r(148) = 0.31, p < 0.001 (Table 8). In the case of grit, the results indicated a significant positive correlation between grit (POE) and L2 WTC, r(148) = . 37, p < 0.001. Grit (COI) on the other hand did not show a significant correlation with L2 WTC, r(148) = . 14, p > 0.078. Regarding the private institute learners, the results indicated significant positive associations between CE, r(121) = 0.31, p < 0.001, COI, r(121) = 0.24, p < 0.007, and POE, r(121) = 0.53, p < 0.001, and with private institutes’ EFL learners’ L2 WTC.

**Table 8:** Spearman’s rho correlation, Grit, CE, and WTC for the public school students and private institute learners

			Public schools	Private institutes
Spearman’s rho	Grit (POE)	Correlation coefficient	0.373**	0.535**
		Sig. (2-tailed)	0.000	0.000
	Grit (COI)	Correlation coefficient	0.145	0.245**
		Sig. (2-tailed)	0.078	0.007*
	CE	Correlation coefficient	0.311**	0.539**
		Sig. (2-tailed)	0.000	0.000*

**Correlation is significant at the 0.01 level (2-tailed)

*Correlation is significant at the 0.05 level (2-tailed)

### Regression analyses

Two separate regression analyses were also performed to estimate the predictive power of L2 girt (POE & COI) and CE for L2 WTC. Table 9 shows information about the regression model as a whole. The three variables together positively correlated with the total score at 0.55, which is almost high. The adjusted *R*2 indicated that the model significantly predicted 29 percent of the variance in the population.

**Table 9:** Multiple regression analysis for the public school students

Model summary				
Model	R	R square	Adjusted R square	Std. error of the estimate
1	0.555^a^	0.308	0.294	0.76180

^a^Predictors: (Constant) CE, Grit

To check the significance of these relationships, the results of ANOVA is required (Table 10). The results indicated that POE and CE significantly predict learners’ WTC (F (37.24) = 3.14, p < 0.001).

**Table 10:** The ANOVA^a^ results for the public school students

Institutes	Model		Sum of squares	Df	Mean square	F	Sig
Public schools	1	Regression	37.245	3	12.415	21.392	0.000^b^
		Residual	83.569	144	0.580		
		Total	120.813	147			

^a^Dependent variable: WTC

^b^Predictors: (constant), CE, Grit

Moreover, the coefficients table (Table 11) showed that POE as the grit’s first component (B = 0.30, p < 0.001) and CE (B = 0.23, p < 0.001) wielded a significant predictive power for public schools’ students L2 WTC. On the other hand, COI did not show any significant predictive power for L2 WTC among learners (B = 0.0.010, p > 0.872).

**Table 11:** Coefficients of grit and CE for the public school students

Coefficients^a^							
	Model		Unstandardized coefficients		Standardized coefficients	T	Sig
			B	Std. error	Beta		
Public schools	1	(Constant)	0.991	0.215		4.610	0.000
		POE	0.307	0.058	0.396	5.269	0.000
		COI	0.010	0.065	0.012	0.161	0.872
		CE	0.233	0.067	0.264	3.458	0.001

^a^Dependent variable: WTC

Table 12 demonstrates that the three variables together positively correlate with the total score at R = s0.720, which is high. *R-*squared (*R*2) shows 51 percent of the variance. The adjusted *R*^2^ in the model reveals that CE and grit could predict 50 percent of EFL learners’ L2 WTC.

**Table 12:** Multiple regression analysis for private institute learners

Model summary					
Private institutes	Model	R	R square	Adjusted R square	Std. error of the estimate
	1	0.720^a^	0.518	0.506	0.72887

^a^Predictors: (constant), CE, Grit

The results of the ANOVA (Table 13) test indicated that POE and CE, significantly predict learners’ WTC (F (66.806) = 3.11, p < 0.001).

**Table 13:** The ANOVA^a^ results for private institute learners

The ANOVA results							
Private institute	Model		Sum of squares	Df	Mean square	F	Sig
	1	Regression	66.806	3	22.269	41.917	0.000^b^
		Residual	62.156	117	0.531		
		Total	128.962	120			

^a^Dependent Variable: WTC

^b^Predictors: (Constant), CE, Grit

The table of coefficients (Table 14) displays that POE as the grit’s first component (B = 0.39, p < 0.001), and CE (B = 0.42, p < 0.001) exerted a significant predictive power over learners’ L2 WTC in the private institutes setting. COI, however, did not wield a significant effect on L2 WTC.

**Table 14:** Coefficients of grit and CE for private institute learners

Coefficients^a^							
Private institutes	Model		Unstandardized coefficients		Standardized coefficients	T	Sig
			B	Std. error	Beta		
	1	(Constant)	0.437	0.303		1.441	0.152
		POE	0.394	0.064	0.434	6.143	0.000
		COI	0.029	0.076	0.025	0.380	0.704
		CE	0.421	0.075	0.412	5.644	0.000

^a^Dependent variable: WTC

## Discussion

This study was heuristic in that it is among the first efforts to compare the relationship between positive individual (grit) and situational (CE) variables and L2 WTC among EFL learners in both public schools and private institutes. The results of the present study, to some extent, replicate but mainly extend previous findings on EFL learners’ L2 WTC. Our approach was consistent with the goal of positive psychology (Seligman & Csikszentmihalyi, 2000) and L2 WTC theory (MacIntyre et al., 1998) to understand the possible links between positive emotions and success (L2 WTC in this case). Several significant points were observed after analyzing the data, which will be discussed in the following sections.

### Public school and private institute learners’ L2 WTC

The descriptive data and the Mann–Whitney U test demonstrated that learners in private institutes enjoy higher levels of L2 WTC compared with public school students. The reasons for this finding can be explained from the socio-educational perspective. First, Iranian public schools and private institutes differ in their teaching methodology. The former syllabus for English in public schools was mainly based on the GTM (Rahimi, 2009). Although the current so-called ‘revised’ version (in effect since 2013) is claimed to be in line with the CLT perspectives, it focuses on inductive grammar teaching, reading comprehension, partial vocabulary instruction, limited speaking exercises in the form of drills (Sadeghi & Richards, 2016), and has failed to promote communicative proficiency among students.

On the other hand, private institutes possess a decentralized system, enabling them to select their own textbooks and methodology. Almost all of the institutes fashion their methodology based on the principles of the communicative competence approach (Zhang & Rahimi, 2014) so as to prepare learners to communicate in both spoken and written modalities (Rahimi & Zhang, 2015). Naturally, the different teaching methodology of the two enterprises can be a justifiable reason why the WTC of the learners would differ in the different contexts.

Second, the majority of classrooms in Iranian public schools are teacher-dominated. This teacher-centered method at schools can deter students’ participation in the classrooms. Zarei et al. (2019) suggested that the dominant teacher-centered style in Iran’s educational system, teachers’ roles in the classroom, and institutional expectations, are debilitating factors concerning WTC. Moreover, in teacher-centered classrooms, pair/group work, which is likely to trigger active student–student interaction in the L2, is ignored to a great extent. Many researchers (e.g., Ahlquist, 2019; Fushino, 2010) have proved group work significantly affects the ease of language use. Private institutes’ classrooms are less teacher-dominated in comparison to public schools, and pair/group work is more common. In Cao and Philp’s (2006) words, group work can easily advantage language learners and give them a sense of security to initiate and participate in communicative acts.

Third, as Robertson et al. (2000) pointed out, culture can affect teachers’ and students’ interactions. Students entering the EFL classrooms for the first time in public schools have already formed a perception of the roles normally held by the teachers based on the cultural norms and their experiences with teachers in other subjects. They are likely to find it natural to hold their teachers in high regard, which brings us to the issue of ‘power distance.’ As Hofstede (2001) puts it, power distance is distributed unequally, yet it is ‘accepted’ and ‘expected.’ Public schools’ classrooms are teacher-centered, where teachers’ role as the dominant authority is accepted and expected. This often hinders a possible yet needed friendly atmosphere in the classrooms, which can enhance students’ willingness to participate in or initiate communication. Dewaele et al. (2019) confirmed that teachers should be supportive, funny, and friendly to enhance the effects of novel and exciting classroom activities.

Regarding item 30 in this study’s questionnaire, which addressed ‘teachers’ friendliness,’ the researchers observed a large mean difference between the learners’ responses in public schools and private institutes. This suggests that learners in private institutes find their teachers friendlier. This is understandable considering the smaller age difference between the EFL learners and their teachers in the private institutes, the less teacher-dominant classrooms, and the employment of exciting activities in this context, which reduces the power distance between teachers and the learners and increases the possibility of classroom communication.

Fourth, Iranian public schools are test-oriented, and performance-based tasks are not a part of the criteria for the final assessment (Farhady & Hedayati, 2009). This is understandable when considering the Iranian public schools’ context. English study for most public schools’ students in Iran is undertaken to meet the need for passing examinations rather than for communicative purposes. A similar situation has been reported in the Chinese context. Peng and Woodrow (2010) found that both EFL teachers and learners prioritize test-related skills such as vocabulary, reading, and writing over speaking in an exam-oriented context. This contrasts with private institutes where performance-based tasks are included as a part of their examination criteria; therefore, learners are more willing to participate in the communication. These possible reasons resonate with those hinted at by Yashima et al. (2017) and Lee and Lee (2020). They realized that inserting performance-based activities in the assessment criteria can enhance students’ WTC in Japanese and Korean contexts, also counted as exam-driven countries.

#### CE and WTC

The results of this study revealed that CE positively correlates and predicts L2 WTC in both contexts. There was, however, a small difference between the perceived CE among learners in these two settings. The EFL learners in public schools experienced CE slightly less than those in private institutes. Adding to the nascent but growing literature focused on CE, these findings bring the important relationship between CE and L2 WTC into the spotlight.

The positive relationship between CE and WTC can be explained in terms of Fredrickson’s (2003) ‘broaden and build’ theory. The ‘broaden’ side of this theory suggests that positive emotions can motivate learners to explore new experiences and seek opportunities for learning more efficiently. In order words, learners would be more willing to participate in communicative activities when they enjoy the classroom. Moreover, through creating active approaches to peers and teachers, positive emotions such as CE can also diminish the effect of negative emotions that act as hindering factors in relation to L2 WTC (Dewaele & Alfawzan, 2018). These findings are in line with those of the prior studies that investigated the possible link between positive emotions and L2 WTC (Khajavy et al., 2018; Khajavy et al., 2021a, 2021b; Jiang & Dewaele, 2019; Peng, 2015).

#### Grit and L2 WTC

Grit (POE) displayed a positive correlation and significant predictive power in relation to L2 WTC in both contexts. By comparing the reported results, one can notice that POE was higher among private institutes’ learners. We can infer from the analysis that grittier students who show constant effort can be more inclined to engage in communication. This is in agreement with the findings of Dörnyei and Ushioda (2013) who explained that success in learning an L2 is highly reliant on learners’ sustained effort. These findings can be reviewed in light of the incremental theory of intelligence (Costa & Faria, 2018; Lindblom, 1950), which explains that intelligence is a malleable quality that can be developed. In this regard, EFL learners with a malleable perspective toward language learning are expected to exert themselves to achieve goals despite setbacks. The findings of other studies that have established positive associations between grit and SLA further confirm this claim. Teimouri et al. (2020) asserted that grittier students are more likely to engage in class discussions than their less gritty peers. In relation to L2 WTC, Lee (2020) also demonstrated that POE is predictive of L2 WTC.

Furthermore, the results suggested no significant predictive power and correlation concerning COI with/for L2 WTC in the public schools’ setting. In the private institutes, however, a significant correlation, but no predictive power was observed between COI and L2 WTC. This finding also implies that learners’ interest in language learning may suffer some wax and wane during the process of language learning, a process which is naturally replete with lengthy and tedious activities. Nevertheless, learners might still work hard to achieve their goals. Similar findings were reported in prior research in other contexts such as Korea (Lee, 2020), Switzerland (Karlen et al., 2019), and China (Feng & Papi, 2020). Credé et al., (2017, p. 502) conclusion that “perseverance is a much better predictor of performance than either consistency or overall grit …” further buttresses our findings regarding COI.

## Conclusion

WTC has been studied from a variety of perspectives, from being examined as a personality trait disposition to a novel situational approach. The results of this study showed that students in public schools suffer a lower level of L2 WTC compared to EFL learners in private institutes. Many researchers have suggested various ways to nurture L2 WTC in EFL classrooms. For instance, Cao and Philp (2006) suggested familiarity with interlocutors, familiarity with the topics, and self-confidence as important factors to increase learners’ WTC. Public schools’ EFL teachers, therefore, should know that although speaking in the target language is not a part of the examination criteria, they can assign grades to this skill. Yashima et al. (2017) and Lee and Lee (2020) confirmed that including performance-based tasks can help students be more willing to participate in the communication acts. Iran’s Education Ministry authorities and curriculum designers should also make changes in the overall English education system so as to reinforce the WTC among EFL learners.

The language learning process can be, at times, tedious and challenging (e.g., participating in communicative activities). EFL teachers should be aware of the importance of grit (POE) and how it can help learners during this process. These teachers should introduce their students to the malleable nature of intelligence and inform them that besides talent, more valuable factors such as diligence and perseverance can help them achieve their goals. According to Keegan (2017), being ‘gifted’ is not the only predictor of success in language learning. In relation to L2 WTC, since many teachers, regardless of where they are teaching, struggle to engage their students in communication, the results of this study, which depicted POE as a positive predictor of L2 WTC, can be of great interest. Teachers can ensure that their learners are familiar with the positive role of POE by giving lectures, introducing successful people, and encouraging them to be persistent to achieve their objectives.

This study revealed the positive link between an external positive emotion (CE) and L2 WTC. EFL teachers, especially in public schools which displayed lower levels of CE, should know that besides boosting students’ L2 WTC (Cheng, 2021; Lee, 2020), positive emotions such as enjoyment can attenuate the hampering effects of negative emotions on L2 WTC (Jin & Zhang, 2018). To promote a positive and enjoyable atmosphere in the EFL classrooms, teachers should be encouraged to reduce the power distance by having a friendly tone, employing exciting activities, and helping learners feel secure enough to start communicating in the target language.

Some limitations need to be taken into account when interpreting the results of this study. First, a close-ended questionnaire to collect the data was employed due to the restrictions imposed by the Covid-19 pandemic at the time of conducting this study and the practicality of online questionnaires at that critical time. This resulted in the deprivation of the present study of any qualitative data. Future studies can use multiple data collection methods such as field observations and interviews to ensure the integrity of the data.

Second, since the researchers intended to compare the results obtained from public schools and with those of private institutes, students with the same level of proficiency in the two contexts had to be selected. Due to the limited curriculum in Iranian public schools, English proficiency among public school students remains low, almost equal to the lowest level of proficiency in private institutes. Therefore, this can limit the generalizability of the findings to other proficiency levels. The present study encourages further subsequent research on public schools’ EFL learners' L2 WTC, which is being ignored to an unfortunate degree. Other investigations with a larger sample size regarding the roles and effects of grit and CE on L2 WTC would be worthwhile and generalizable to Iranian EFL learners.

Third, it is worth mentioning that Iran is a multilingual and multicultural country. The present study was conducted from a socio-educational perspective. Therefore, future studies examining the same variables regarding cultural issues would yield more sustainable results.

Fourth, the effects of gender on these variables were not addressed in this study and, to our best understanding, not at all in the Iranian context. Further research can compare the variances due to gender differences.

## Data Availability

The raw data supporting the conclusions of this article will be made available by the authors, without undue reservation.

## Abbreviations

CE: Classroom enjoyment
COI: Consistency of interest
EFL: English as a foreign language
FLCA: Foreign language classroom anxiety
FLE: Foreign language enjoyment
FLP: Foreign language performance
L2 WTC: Willingness to communicate in a second language
L2: Second language
POE: Perseverance of effort
SLA: Second language acquisition
WTC: Willingness to communicate
